# Sex-specific alterations in the gut and lung microbiome of allergen-induced mice

**DOI:** 10.3389/falgy.2024.1451846

**Published:** 2024-08-15

**Authors:** Carolyn Damilola Ekpruke, Rachel Alford, Dustin Rousselle, Maksat Babayev, Shikha Sharma, Erik Parker, Kyle Davis, Christopher Hemmerich, Douglas B. Rusch, Patricia Silveyra

**Affiliations:** ^1^ Department of Environmental and Occupational Health, School of Public Health, Indiana University, Bloomington, IN, United States; ^2^ Department of Epidemiology and Biostatistics, Biostatistics Consulting Center, School of Public Health, Indiana University, Bloomington, IN, United States; ^3^ Center for Genomics and Bioinformatics, Indiana University, Bloomington, IN, United States; ^4^ Division of Pulmonary, Critical Care, Sleep, and Occupational Medicine, Department of Medicine, School of Medicine, Indiana University, Indianapolis, IN, United States

**Keywords:** lung microbiome, gut microbiome, sex differences, allergic asthma, lung inflammation

## Abstract

**Introduction:**

Recent evidence has demonstrated that the microbiome is a driver of the underlying pathophysiological mechanisms of respiratory disease. Studies have indicated that bacterial metabolites produced in the gut and lung can impact lung inflammation and immune cell activity, affecting disease pathology. Despite asthma being a disease with marked sex differences, experimental work linking microbiomes and asthma has not considered the sex variable.

**Methods:**

To test the hypothesis that the lung and gut microbial composition impacts allergic lung inflammation in a sex-specific manner, we evaluated lung and gut microbiome alterations in a mouse model of allergic inflammation and assessed their association with lung function and inflammation phenotypes. For this, we exposed male and female adult C57BL/6J mice intranasally to 25 µg of a house dust mite extract mix (HDM) daily, or phosphate-buffered saline (PBS) as control, for 5 weeks (*n* = 4–6/group). DNA from fecal pellets collected before and after the 5-week treatment, and from lung tissue collected at endpoint, was extracted using the ZymoBIOMICS®-96 MagBead DNA Kit and analyzed to determine the 16S microbiome via Targeted Metagenomic Sequencing.

**Results:**

The HDM treatment induced a sex-specific allergic inflammation phenotype with significantly higher neutrophilia, lymphocytosis, inflammatory gene expression, and histopathological changes in females than males following exposure to HDM, but higher airway hyperresponsiveness (AHR) in males than females. In addition, sex-specific lung gene expression and associated pathways were identified HDM mix after challenge. These changes corresponded to sex-specific alterations in the gut microbiome, where the *Firmicutes* to *Bacteroidetes* ratio (F:B) was significantly reduced in fecal samples from only male mice after HDM challenge, and alpha diversity was increased in males, but decreased in females, after 5-weeks of HDM treatment.

**Discussion:**

Overall, our findings indicate that intranasal allergen challenge triggers sex-specific changes in both gut and lung microbiomes, and induces sex-specific lung inflammation, AHR, and lung inflammatory gene expression pathways, suggesting a contribution of the lung-gut axis in allergic airway disease.

## 1 Introduction

Asthma is a common respiratory disease, characterized by chronic airway inflammation and airflow limitation, that affects people at all life stages. It is a multifactorial disease that can result from interactions between genetic, environmental, and lifestyle factors (1), as well as epigenetic contributions (2). The prevalence of asthma has been increasing in recent decades. The most recent Global Burden of Disease (GBD) study by The Global Asthma Network estimated that asthma affected around 262 million people and that over 400,000 deaths resulted from the effects of the disease in 2019 alone (3). Notably, the prevalence and severity of asthma symptoms are reported to be greater among adult women when compared to men (4). Many clinical and basic science studies have attributed this disparity to the effect of circulating sex hormones on the physiological and immunological functions of the lung (5). The interaction between hormones and inflammation has been previously implicated in the pathogenesis of asthma (6). However, it remains unclear to what extent sex influences other mediators of respiratory disease and inflammation, such as the microbiome.

Pathophysiology research has suggested that the microbiome is a major driver of disease and illness. The microbiome is the trillions of microbes, including bacteria, viruses, and fungi, that reside in communities within and on the human body, providing metabolic and immune functions (7, 8). These microorganisms are highly influential on the health of their host, and alterations in the composition and diversity of the microbiome have been associated with a wide range of diseases; including autoimmune disorders, cancers, and lung diseases (9). These include tryptophan metabolites which can induce cytokine expression, causing the production of antibacterial peptides by acting as aryl hydrocarbon receptor ligands (10). Immunity and Intestinal barrier function can also be influenced through metabolism of short-chain fatty acids and bile acids (11).

Microbial profiling using the 16S microbiome is a common technique for studying microbial communities in various environments, including in the gut and respiratory tract (7). The 16S microbiome is based on sequencing the 16S ribosomal RNA gene, which is present in all bacteria and is highly conserved across species (12). The technique is used to identify and classify bacterial taxa based on their unique 16S rRNA sequences. Several studies have suggested that gut microbiome alterations may contribute to asthma development and exacerbation (13, 14). Growing evidence suggests that dysbiosis, or disruption, of gut microbiota, is an important contributor to the pathophysiology of inflammatory lung disease (15, 16). *Bacteroidetes* and *Firmicutes* are the predominant phyla of bacteria found in the gut microbial community; while *Bacteroidetes*, *Firmicutes*, and *Actinobacteria* are found to predominate in a healthy lung (17, 18). The gut and lungs are anatomically distinct, both organs have anatomical origins and complex pathways that reinforce the potential for the existence of the gut-lung axis (19, 20). Several microbiological and epidemiological studies report the contributions of the communities of the gut microbiome, in addition to the lung microbiome to the development of asthma in children. The gut-lung axis is the bidirectional communication between the gut and the lungs (21). It is an emerging field of research that seeks to understand the complex interactions between the gut microbiome, the mucosal immune system, and lung health. Much evidence suggests that bacterial metabolites produced in the gut can impact lung inflammation and immune cell activity and that the microbiome plays a role in the pathology of asthma, but the mechanisms involved are not clear. The mechanisms by which gut microbiota has been shown to regulate immune responses in the lungs are varied, including the “production of host-accessible metabolites, penetration of mucosal barriers by bacteria and/or toxins, regulation of hematopoiesis or circulating hormones and cytokines, and direct stimulation of migrating immune cells” (22).

Conversely, lung diseases can also affect the gut microbiome. For example, individuals with chronic obstructive pulmonary disease (COPD) also display altered gut microbiome composition and diversity, with an overgrowth of pathogenic bacteria, when compared to healthy individuals (15, 23). Overall, the gut-lung axis represents an important area of research in the respiratory medicine field. A better understanding of the interactions between the gut microbiome, the immune system, and lung health will contribute to the development of improved disease management and prevention strategies that can be used to decrease the global burden of asthma. The current study investigated the sex-specific effects of an environmental allergen challenge on the community and structure of the gut and lung microbiomes’ composition, diversity, and abundance using a mouse (C57BL/6J) model.

## 2 Materials and methods

### 2.1 Animal model

The animal study protocol (Protocol 21-012) was approved by Indiana University Bloomington's Institutional Animal Care and Use Committee (BIACUC). This study used an inbred strain, C57BL/6J, mouse model originally from Jackson Laboratories (Bar Harbor, ME), but bred and maintained in-house. Males and Females of 8–10-week-old littermates were randomly assigned (*n* = 4–6/group) to control or treatment groups. Those assigned to the experimental group were intranasally exposed to 25 µg of the allergen extract from house dust mite mix of *D. pteronyssinus*, and *D. farinae* mix (HDM) (Citeq biologics, Groningen, Netherlands) suspended in 50 µl of phosphate-buffered saline (PBS) daily (5 days/week) for 5 total weeks after a light anesthesia with 5% isoflurane using the SomnoSuite device (Kent Scientific) to induce phenotypic allergic airway inflammation, a physiologically relevant model to study mechanisms of inflammation similar to those occurring in some types of human asthma. Those assigned to the control group were intranasally exposed to 50 µl of PBS daily throughout the same treatment period.

### 2.2 Fecal pellet collection and processing

Fecal pellet samples were collected from each mouse 1–3 days before the commencement of treatment to measure pre-exposure bacterial composition. Once treatment began, fecal pellet samples were collected from each mouse once weekly until the end of the five-week treatment period. Collections were labeled, weighed, and stored in 2 ml tubes at −80℃ until sent for processing. Fecal pellet samples collected during week 0 (pre-exposure) and week 5 (final exposure) were submitted to Zymo Research, to be processed with ZymoBIOMICSⓇ Service: Targeted Metagenomic Sequencing (Zymo Research, Irvine, CA). DNA was extracted to a 50 µl elution volume using ZymoBIOMICSⓇ-96 MagBead DNA Kit with ZymoBIOMICSⓇ Microbial Community Standard as a positive control. Afterward, the sample was prepared for targeted sequencing with Quick-16STM NGS Library Prep Kit using customized primers from the Quick-16STM Primer Set V3-V4. Final products from polymerase chain reactions (PCR) performed in real-time PCR machines were quantified with qPCR fluorescence readings and pooled together based on equal molarity to prepare a sequencing library. ZymoBIOMICSⓇ Microbial Community DNA Standard was used as a positive control for each target and negative controls were included. The final library was cleaned up using the Select a-Size DNA Clean & Concentrator™ then quantified with TapeStationⓇ (Agilent Technologies, Santa Clara, CA) and QubitⓇ (Thermo Fisher Scientific, Waltham, WA). The library was sequenced on IlluminaⓇ MiSeq™ with a v3 reagent kit (600 cycles). The sequencing was performed with a 10% PhiX spike-in.

### 2.3 Airway hyperresponsiveness assessment through methacholine challenge

At 72 h after the last exposure, Mice were anesthetized with intraperitoneal administration of Ketamine/xylazine and tracheostomized using an 18-gauge cannula, after which the mice were connected to the rodent ventilator system (flexiVent, SCIREQ, Montreal, Canada). To paralyze the mice muscle, we used pancuronium bromide (10 mg/g). The settings used to ventilate the animals include a positive end-expiratory pressure (PEEP) of 3cmH2O, a tidal volume of 10 ml/kg, and a maximum inflation pressure of 30cmH2O. We then administered Methacholine (MCh) (100 mg/ml) using a nebulizer. Resistance (Rrs), Elastance (Ers), Conducting airway resistance (Rn), Tissue resistance (G), and Tissue elastance (H) were determined after methacholine challenge in all the groups (HDM and PBS-treated groups).

### 2.4 Bronchoalveolar lavage fluid (BALF) immune cells population analysis

Mice were tracheostomized and 1 mm EDTA/PBS solution was used to wash the lungs repeatedly until a solution of 2.5 ml was obtained from each mouse. BALF samples were centrifuged (1,300 rpm for 10 min). Cell pellets were resuspended in 500 ml of 1 mm PBS/EDTA, and 100 ul of the cell suspension was placed in a cytospin slide for differential cell counting using HEMA 3 staining (Fisher # 122-911 protocol HEMA 3 stain set). Cell differentials were counted at least 5 image frames at 20×magnification per slide by three investigators blinded to treatment.

### 2.5 Lung tissue collection and processing for lung microbiome analysis

For the lung microbiome study, whole lung tissues were collected from 8-to-10-week-old C57BL/6J male and female mice (*n* = 4/treatment group) upon sacrifice, and submitted to the University of North Carolina at Chapel Hill Microbiome Core Facility. The lung samples were then collected in a 2 ml tube containing 200 mg of 106/500 μm glass beads (Sigma, St. Louis, MO) and 0.5 ml of Qiagen PM1 (Valencia, CA) buffer was homogenized by mechanical lysis for 40 min. Following 5 min of centrifugation, 0.45 ml of supernatant was aspirated and then transferred into a new tube containing 0.15 ml of Qiagen IRS solution. The suspension was incubated at 4°C overnight. After another 5-minute centrifugation, the supernatant was aspirated and transferred to deep well plates containing 0.45 ml of Qiagen binding buffer supplemented with Qiagen ClearMag Beads. The DNA was purified using the automated KingFisherTM Flex Purification System and eluted in DNase-free water. Total genomic DNA was extracted using RNeasy® PowerMicrobiome® Kit (Qiagen, Valencia, CA) and purified using the automated KingFisher™ Flex Purification System. 16S rRNA gene fragments were amplified using primers F515/R806 to target the V4 region of the bacterial gene 1, 2 in addition to the use of 2× KAPA HiFi HotStart ReadyMix (KAPA Biosystems, Wilmington, MA). Each 16S amplicon was purified using the AMPure XP reagent (Beckman Coulter, Indianapolis, IN). The DNA was then denatured with NaOH, diluted with hybridization buffer, and heat-denatured before loading on the MiSeq reagent cartridge (Illumina, San Diego, CA) and on the MiSeq instrument (Illumina, San Diego, CA). Automated cluster generation and paired–end sequencing with dual reads were performed according to the manufacturer's instructions. Bioinformatics microbiome analyses were conducted as described below.

### 2.6 Lung tissue collection for histological analysis

For lung histology, using another set of mice following the same treatment protocol, mice were anesthetized with ketamine and blood was collected to exsanguinate the animals for lung perfusion. Each mouse chest cavity was opened, the trachea cannulated and 5 µl formalin was placed in a syringe and allowed to perfuse in the lung by gravity. After which, the lung was fixed in formalin overnight. At 24 hrs, the lung was transferred into 70% ethanol solution, then each lobe of the lung was sectioned into half and stained with Alcian blue & Periodic Acid-Schiff (AL-BI-PAS), and Hematoxylin-eosin (HE- collagen). The histology slides were scored using the method used in the previous study (24). All tissues were examined by three investigators blinded to treatments.

### 2.7 Differentially expressed genes (DEGs) assessment in lung tissue

Lung tissues were harvested and pulverized, and RNA was extracted using the Direct-zol RNA Miniprep plus kit (Zymo Research Corporation, Irvine, CA). A Nanodrop ND-2000 spectrophotometer (Thermo Scientific Wilmington, DE, USA) was used to analyze the concentration and quality of the RNA extracted in the different groups. Using targeted sequencing-based RNA expression, the mice RNA (*n* = 3/group) was analyzed, and the result was generated from TempO-Seq. assays (Arctoris Ltd. Oxfordshire, UK).

### 2.8 Ingenuity pathway analysis (IPA)

The QIAGEN Ingenuity Pathway Analysis (QIAGEN Inc., https://digitalinsights.qiagen.com/IPA) (25) was used to construct networks of DEGs in all the groups and to visualize the genes’ associations with canonical pathways implicated in asthma and lung inflammation.

### 2.9 Microbiome analysis

For gut and lung microbiome analysis, unique amplicon sequences were inferred from raw reads using the Dada2 pipeline; additionally, chimeric sequences were removed using the Dada2 pipeline. Taxonomy assignments were performed using Uclust from Qiime v.1.9.1 with the internally designed and curated, Zymo Research Database, used as a reference. Alpha-diversity and beta-diversity analyses were performed with Qiime v.1.9.1. Sequencing output from the Illumina MiSeq PE250 platform was converted to fastq format and demultiplexed using Illumina Bcl2Fastq 2.18.0.12. The resulting reads were processed using QIIME 2. Index and linker primer sequences were trimmed, and the reads were processed with DADA2 through QIIME2 including merging paired ends, quality filtering, error correction, and chimera detection. Each read was then assigned to the GREENGENES database. *Firmicutes/Bacteroidetes* ratio (F: B) was calculated using GraphPad Prism version 9. The raw amplicon sequence variant (ASV) output from QIIME 2 was used to generate stacked taxa bar plots. Beta diversity analysis is displayed by Nonmetric Multidimensional Scaling (NMDS) plots, using Bray-Curtis distances between samples. This method calculates differences between samples based on the presence or abundance of bacterial ASVs, which is visualized as the distance between samples in the figure. The distances are then used to generate plots in N-dimensional space where samples with smaller distances between them are closer together and those with larger distances are further apart. Bray Curtis was chosen because it is sensitive to differences in relative abundances. The NMDS model then maneuvers the points to where there is the best fit between points being close enough and far enough away from each other. Those points are then given symbols based on their identity and overlaid with the other data to see how similar/different our clusters of samples are based on treatment, time, and sex, where closer clusters indicate more similar samples.

### 2.10 Statistical analysis

For AHR, immune cell counts, and lung histology, the average values of Rrs, Ers, Rn, G, and H from the methacholine challenge, alongside the prepared slides for both the immune cell analysis and lung histology were examined by three blind investigators and were collated, using the GraphPad prism 10.0.2. Data were expressed in mean ± SEM. 2-way ANOVA was used to compare the mean effect of sex and treatment in the different groups and results were statistically significant at a *P*-value of <0.05. For microbiome data, the raw ASV was used for permutational ANOVA (PERMANOVA), and ANOVA-like differential expression (ALDEx) analyses in R, using the phyloseq (26), genefilter (27), tidyverse (28), vegan (29), ANCOMBC (30), and ALDEx2 (31) packages, and for generating Bray-Curtis NMDS plots. Finally, for differential gene expression analysis, we used the DESeq2 package (version 1.40.2) in R/Bioconductor (R version 4.3.1) (32). Heatmaps were generated using the R Bioconductor package heatmap (version 1.0.12). Significant differentially expressed genes (DEGs) were determined using a *P* adj. <0.05. Venn diagrams were generated using the R package Venn Diagram (version 1.2.2). UpSet plots were generated using Complex Upset (version 1.3.3) and UpSetR (version 1.4.0).

## 3 Results

### 3.1 Sex differences in air hyperresponsiveness

**Figure 1:**
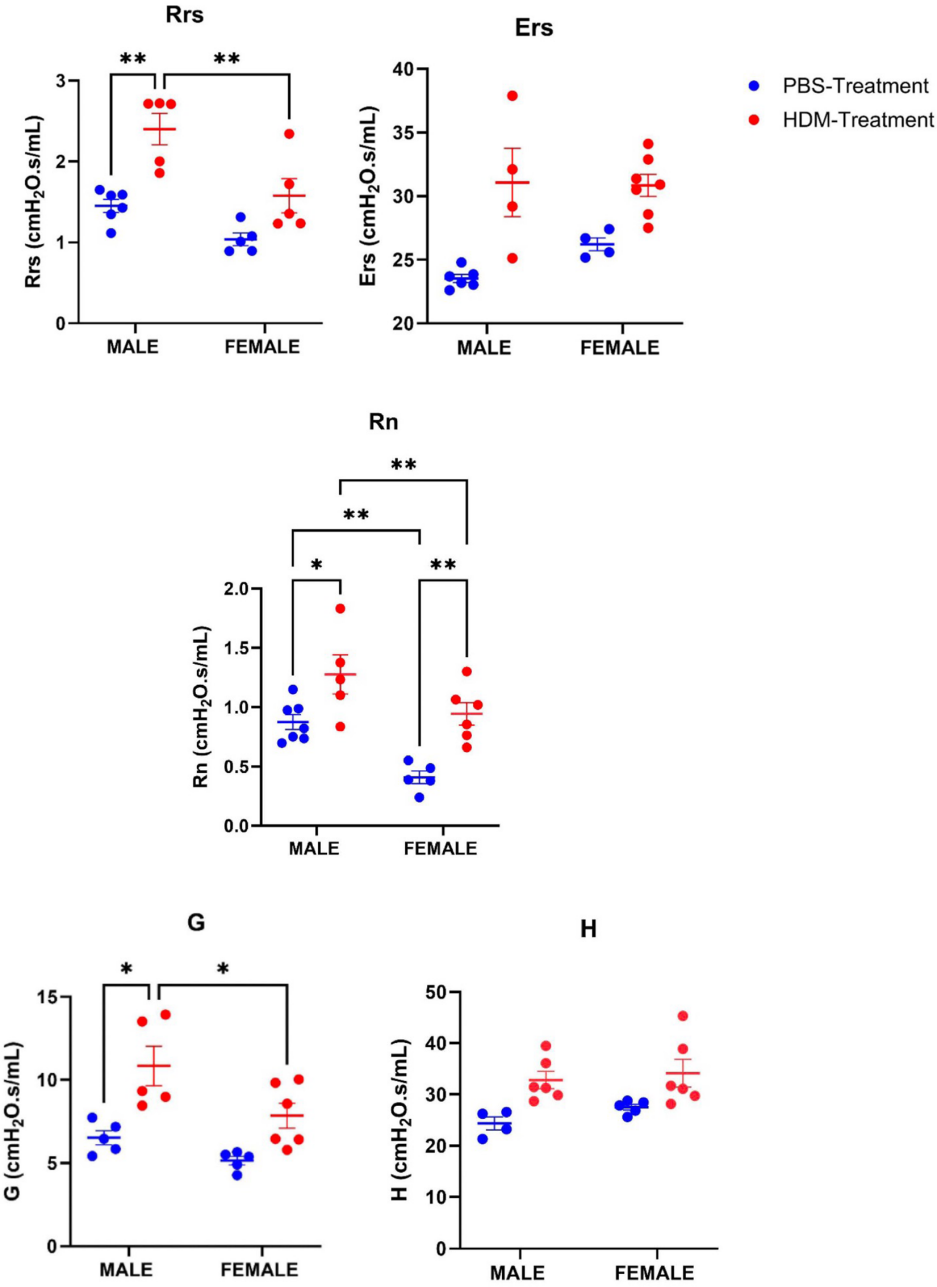
Sex differences in airway hyperresponsiveness following 5 weeks HDM or PBS exposure. Assessing sex differences in airway hyperresponsiveness using the lung parameters, Total airway resistance (Rrs), Elastance (Ers), Tissue elastance (H), Conducting airway resistance (Rn), and Tissue damping (G) in male and female mice (C57BL/6J) after Methacholine (100 mg/ml) challenge following exposure to PBS or HDM. Data represented as mean ± SEM of at least 4-7 mice per treatment group. *P < 0.05; **P < 0.001, ***P < 0.0001, ****P < 0.00001.

AHR was assessed in both male and female mice treated with the HDM mix of *D. pteronyssinus*, and *D. farinae* via methacholine challenge (100 mg/ml). The average values of Rrs, Ers, Rn, G, and H from the methacholine challenge in all the groups were analyzed and the values were seen to increase in the HDM-treated groups compared to the PBS-treated groups at *P* value <0.05 (Figure 1) at the end of the study. Rrs, Rn, and G were significantly increased in the male HDM-treated group compared to the female group. However, Ers and H were not significantly different in the male and female HDM-treated groups.

### 3.2 Sex differences in BALF immune cell populations

**Figure 2:**
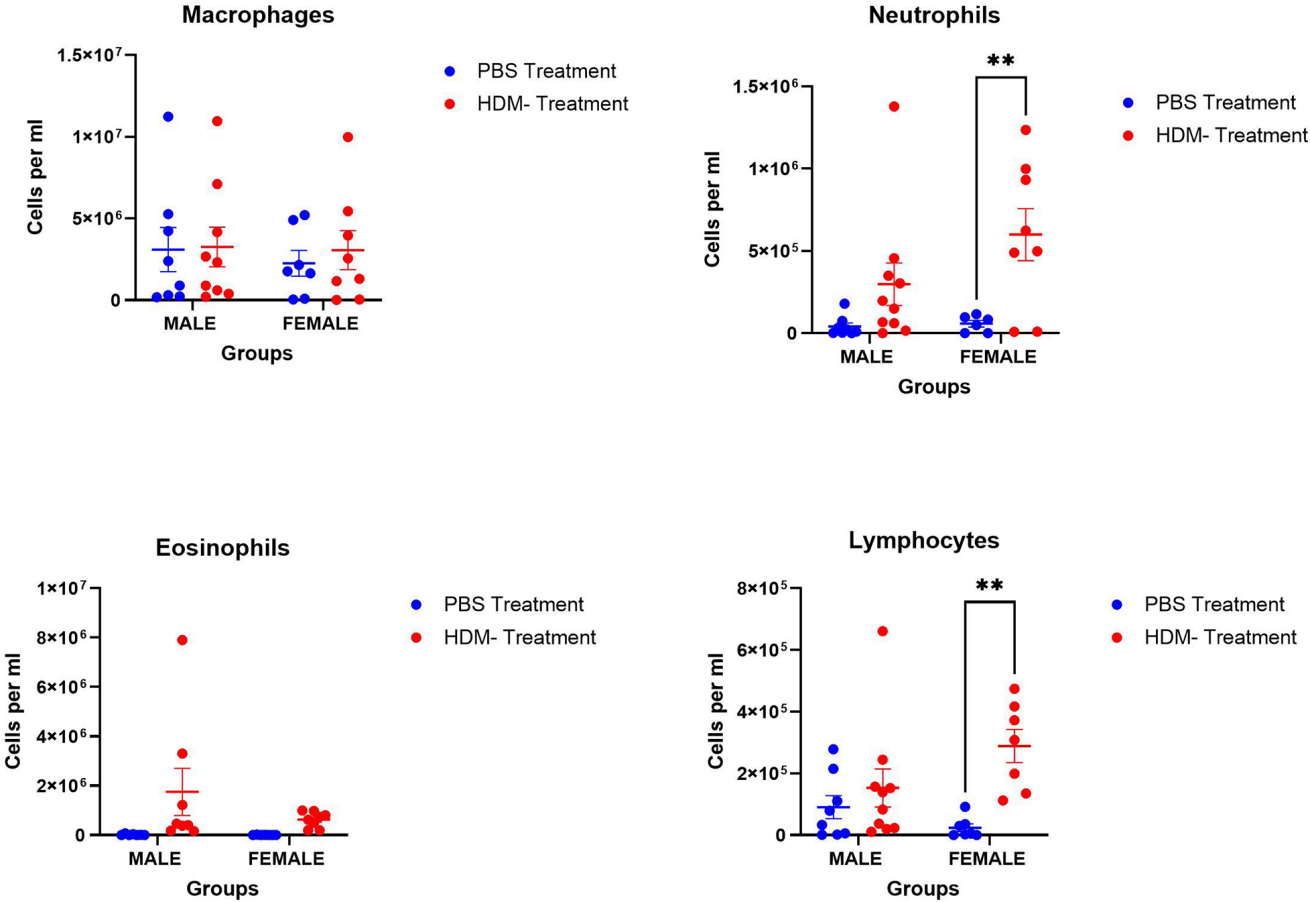
Sex differences in immune cell population in the bronchoalveolar lavage fluid following 5 weeks HDM or PBS exposure. Assessing sex differences in the population of immune cells, macrophages, neutrophils, eosinophils, and lymphocytes in male and female mice (C57BL/6J) exposed to PBS or HDM. Data represented as mean ± SEM of at least 5-9 mice per treatment group. *P < 0.05; **P < 0.001.

BALF neutrophilia and lymphocytosis were observed in the HDM mix-treated groups, although these were significantly higher in female mice (Figure 2). No significant differences were found in the alveolar macrophages and eosinophil counts among the experimental groups.

### 3.3 Sex differences in histopathological changes

**Figure 3:**
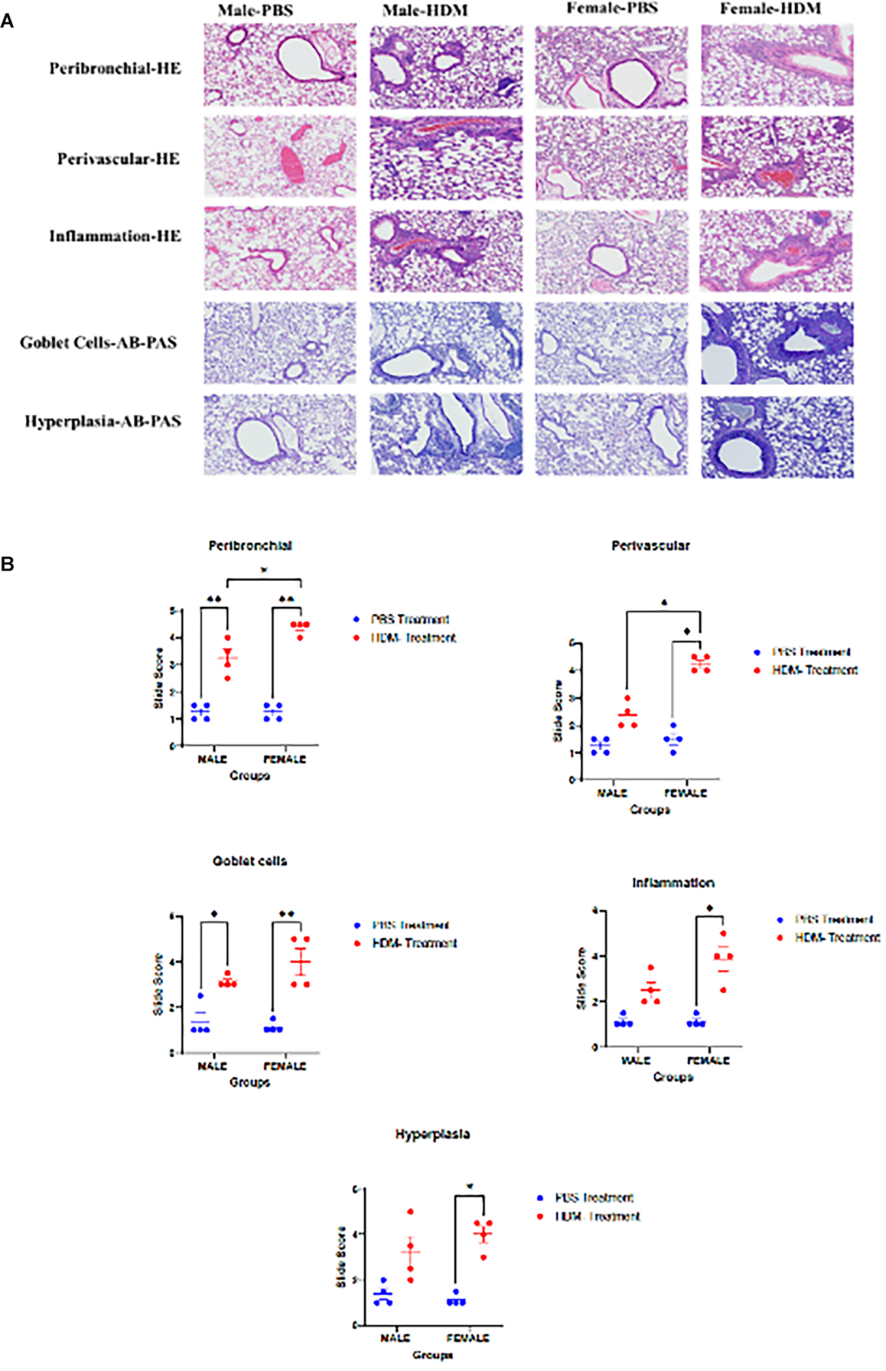
Sex differences in the histopathological analyses of lung tissue following 5 weeks HDM or PBS exposure. (**A**) Histological images of lung tissues stained with Hematoxylin and eosin (HE) or Alcian Blue and Periodic Acid Schiff (AB-PAS) stains to show the degree of peribronchial and perivascular inflammation, inﬂammation score, goblet cells, and hyperplasia. 10× magniﬁcation images showing lung tissues of PBS-treated and HDM-treated male and female (C57BL/6J) mice at 5 weeks and 72 h of the study (*n* = 4/group). (**B**) Graphs representing Slide scoring for each of the groups. Data represented as mean ± SEM of 4 mice per treatment group. *P < 0.05; **P < 0.001.

Histopathological examination of lung tissues showed changes in peribronchial and perivascular inflammation, goblet cells, and hyperplasia in HDM mix-treated mice compared to the PBS-treated group (Figure 3). While peribrochial inflammation and goblet cells were higher in both male and female mice exposed to HDM, perivascular inflammation, inflammation scores, and hyperplasia were only significant in females exposed to HDM vs. PBS. Overall, the female group exposed to HDM displayed a stronger inflammatory phenotype than the male exposed group.

### 3.4 Sex differences in lung gene expression following HDM exposure

**Figure 4:**
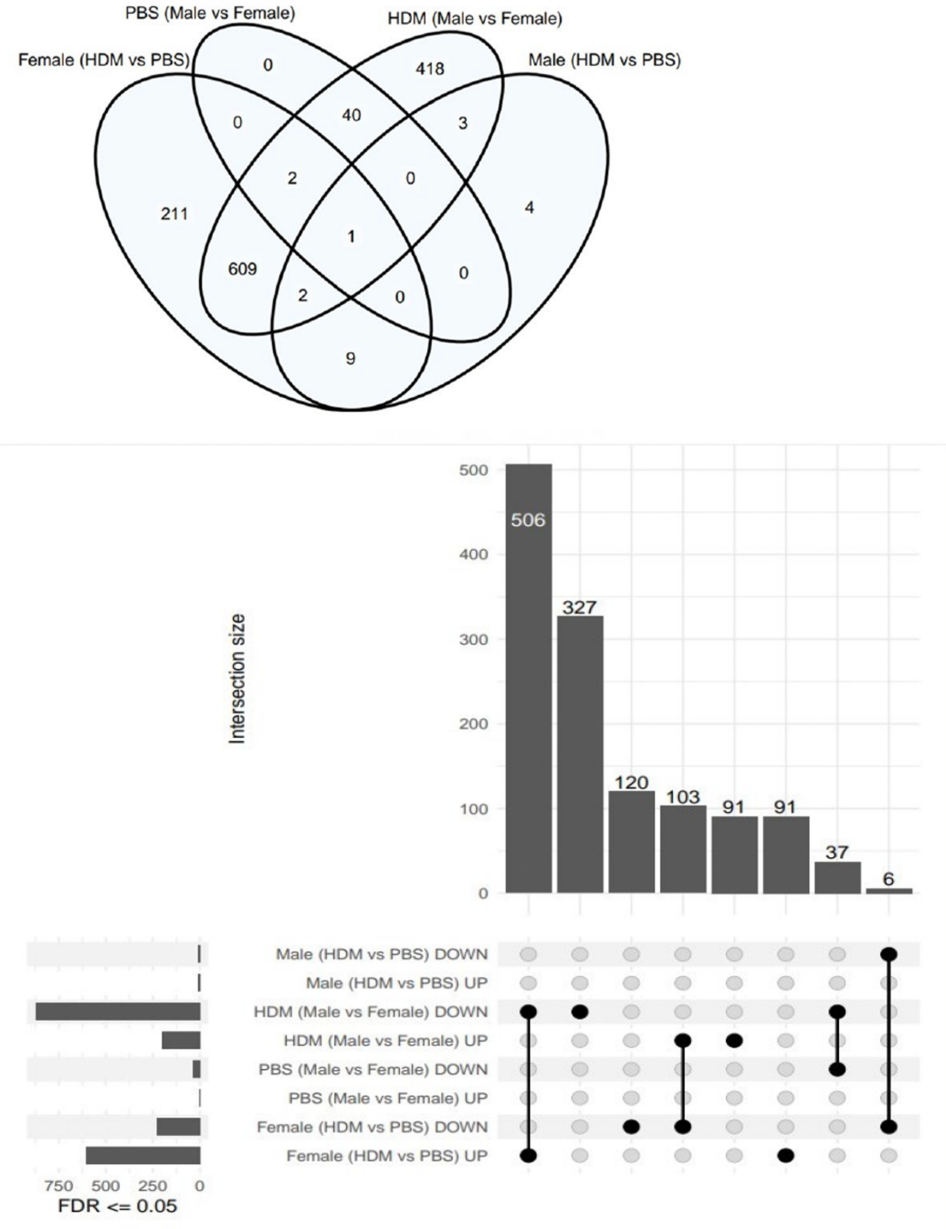
Total genes upregulated or downregulated in the male and female mice following 5 weeks of HDM or PBS exposure. Venn diagram showing the number of unique and shared genes differentially expressed in the lungs of HDM-treated and PBS-treated male and female mice (*n* = 4/ treatment group).

**Figure 5:**
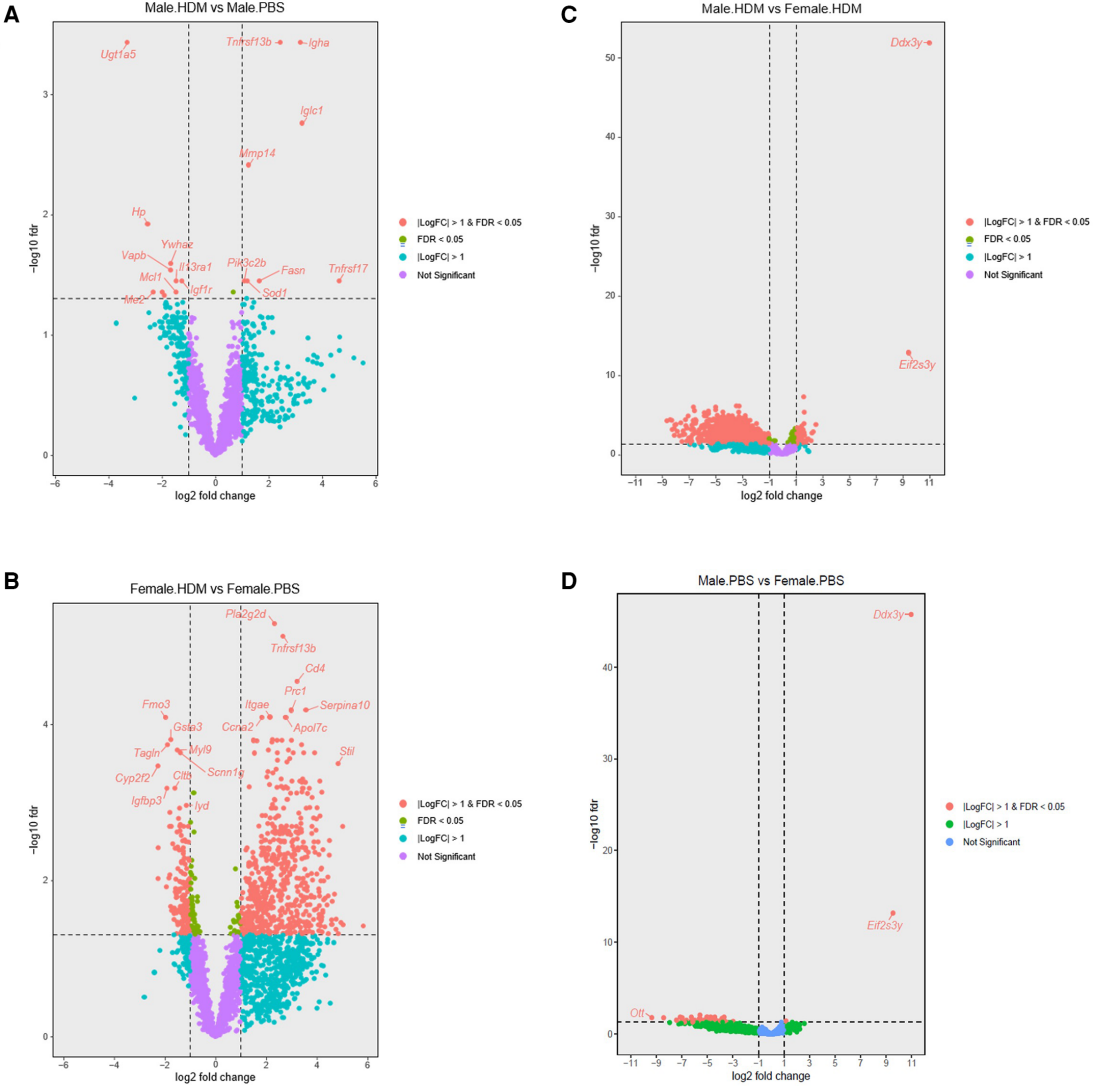
Volcano plots showing sex differences in differentially expressed genes among groups. (**A**) Volcano plot showing differentially expressed genes (DEGs) in the HDM-treated over PBS-treated male mice (*n* = 4). (**B**) DEGs in the HDM-treated over the PBS- treated female mice (*n* = 4). (**C**) DEGs in male and female mice treated with PBS for 5 weeks (*n* = 4). (**D**) DEGs in HDM-treated male and female mice (*n* = 4).

Using the Tempo-seq, a comprehensive gene expression analysis was performed in the lung samples of HDM-treated and PBS-treated male and female mice (Figures 4, 5). Overall, 2,865 differentially expressed genes (DEGs) were observed in the experimental groups. In PBS-treated mice, 43 DEGs were found in males vs. females at FDR <0.05, of which 40 were downregulated (top 10: *Tnfrsf17, Oosp1, Ell3, Gm5128, Magedi, Nlgn3, Itpr1, Tcerg1, Ott, Sprr2a3*), and only 3, *Ddx3y*, *Eiy2s3y*IY2S3Y and Tnnt2 were upregulated. In the HDM-treated mice, 1,075 DEGs were revealed in male vs. female mice, of which 200 were upregulated (top 10: *Ddx3y, Eif2s3y, Klf2, Scnn1 g, Gsta3, Hba-a2, Cyp2b10, Etfb, Htra1, Rela*) and 875 were downregulated (top 10*: Prc1, Kif20a, Nlgn3, Kif2c, Stxbp1, Myh3, Il11, If135, Prune, Pogk*) (Figures 4, 5). When comparing HDM-treated male mice over PBS-treated male mice, only 19 DEGs were revealed, 9 of them upregulated, *Tnfrsf13b, Igha, Iglc1, Mmp14, Pik3c2b, Tnfrsf17, Sod1, Fasn, Itgb5*, while 10 of them downregulated, *Ugt1a5, Hp, Ywhaz, Vapb, Il13ra1, Igf1r, Me2, Ndufs2, Mcl1, Cul1*. Interestingly, in the females, HDM-treated mice showed 834 DEGs (604 upregulated and 230 downregulated) vs. PBS-treated ones. Overall, between male and female mice, a total of 851 DEGs were found in HDM-treated over PBS-treated. Of these, 822 DEGs were in females (599 upregulated and 223 downregulated), and 7 DEGs (3 upregulated and 4 downregulated) in males. However, 12 DEGs (6 upregulated and 5 downregulated) were overlapped between the two groups, while only one gene (*SOD1*) was upregulated in males and downregulated in females.

### 3.5 Sex differences in lung gene expression pathways

Ingenuity pathway analysis (IPA) revealed that the top canonical pathways were B-cell activating factor signaling, *Tnfs, PI3k/Akt* signaling, *Tr/Rxr* activation, and estrogen receptor signaling between HDM and PBS-treated mice. *Pd98059, wortmannin, growth hormone, Nr3c1, and Ly294002* were the top upstream regulators while *Tcf7, Lrig1, Zm306416, Bibx1522,* and *Sema3d* were the top casual network revealed in this group of animals. Interestingly, Immunological diseases and inflammatory responses were among the diseases and disorders associated with HDM-treated male mice over those treated with PBS. Most importantly, significant DEGs, related to inflammatory responses in this group, were *Itgb5, Tnfrsf13b, Mmp14, Sod1, Igf1r, Mcl1,* and *Uwhaz*.

One of the top canonical pathways revealed in HDM-treated female mice over PBS-treated was the *Tp53* pathway. *Rara, I-asparaginase sesaminol, Phox2a, Nr5a2*, *Tnf, Il25, Tp53,* and *IlL6* were the top upstream regulators. Organismal injury and abnormalities were part of the top diseases revealed to be associated with this group. Most importantly, the molecular and cellular functions revealed in this group were cell morphology cellular function/maintenance, and *Pxp/Rxr* activation. Significant DEGs related to inflammatory responses in this group were *Cryba1, Dbh, Tnfrsf17, Trim71, P2ry6, Cerk, Ccl11, Cdk5r1, Il12b, Grn,* and *Npm1*.

**Figure 6:**
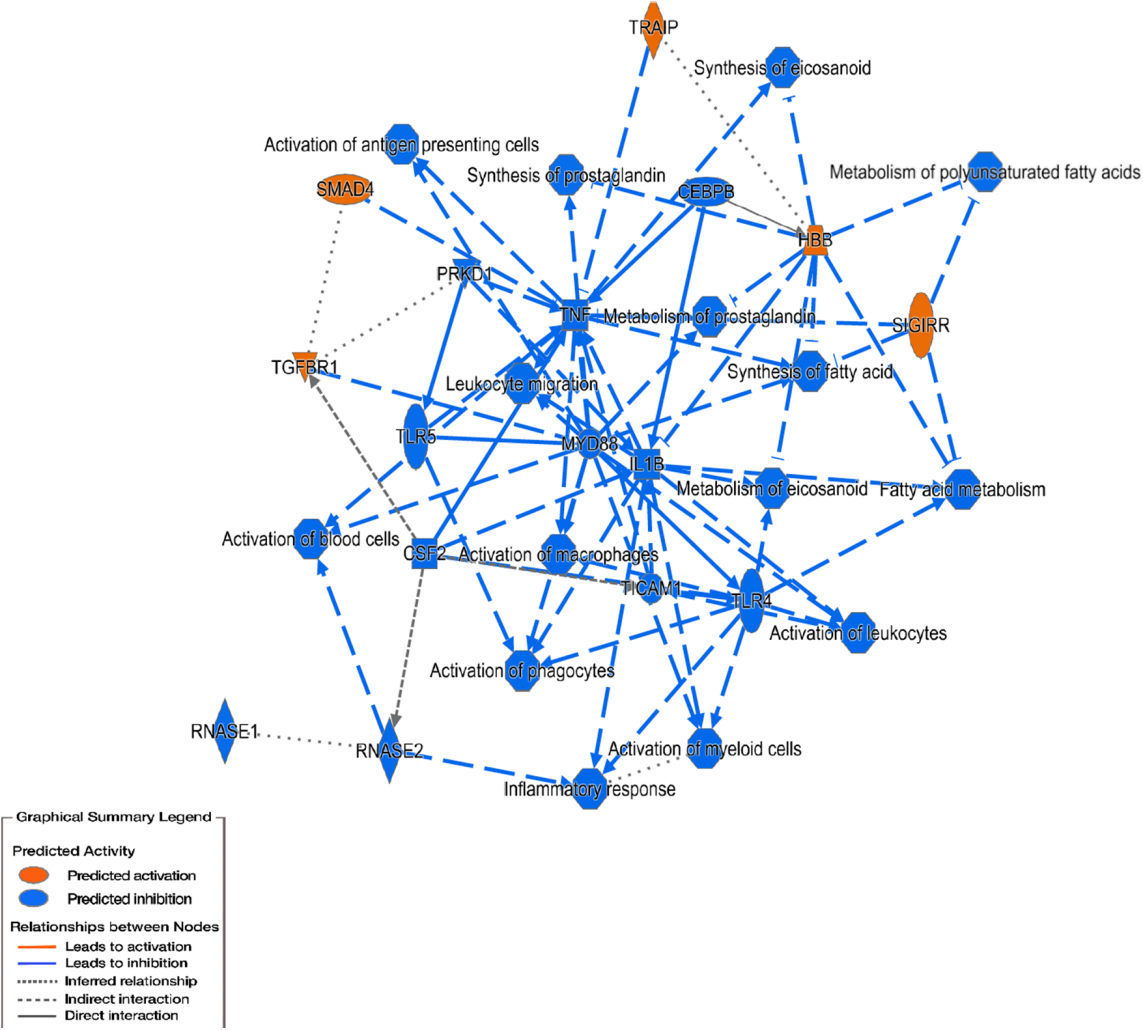
Networks analysis predicted by IPA to be activated or inhibited in the HDM-treated males vs. females. Inflammatory response pathway shown to be inhibited in the male HDM-treated group over the female group. Blue lines represent predicted downregulation.

IPA also showed *Tp53, Tnf, beta-estradiol lipopolysaccharide, and Tgfb1* as the top upstream regulators in the HDM-treated males over the HDM-treated females. Inflammatory response, Endocrine system disorders, and organismal injury/abnormalities were the top diseases and disorders associated with this group (Figure 6). Also, *Smad4, Yap1, Pr0s1, Igfbp3, Sell, Sod1, Sqstm1, Vegfa, Ace, Vwf, Nfe2l2, Vim, Gadd45b, Ghr, Creg1, Rela, Ikbkg, Scp2, Ifngr1, Hsp90b1, Kdr, Tcf4, Bcl2li, Prkca* were DEGs related to inflammatory responses that were significantly expressed in the male HDM-treated group vs. females.

### 3.6 Gut and lung microbiome changes in response to HDM exposure

**Figure 7:**
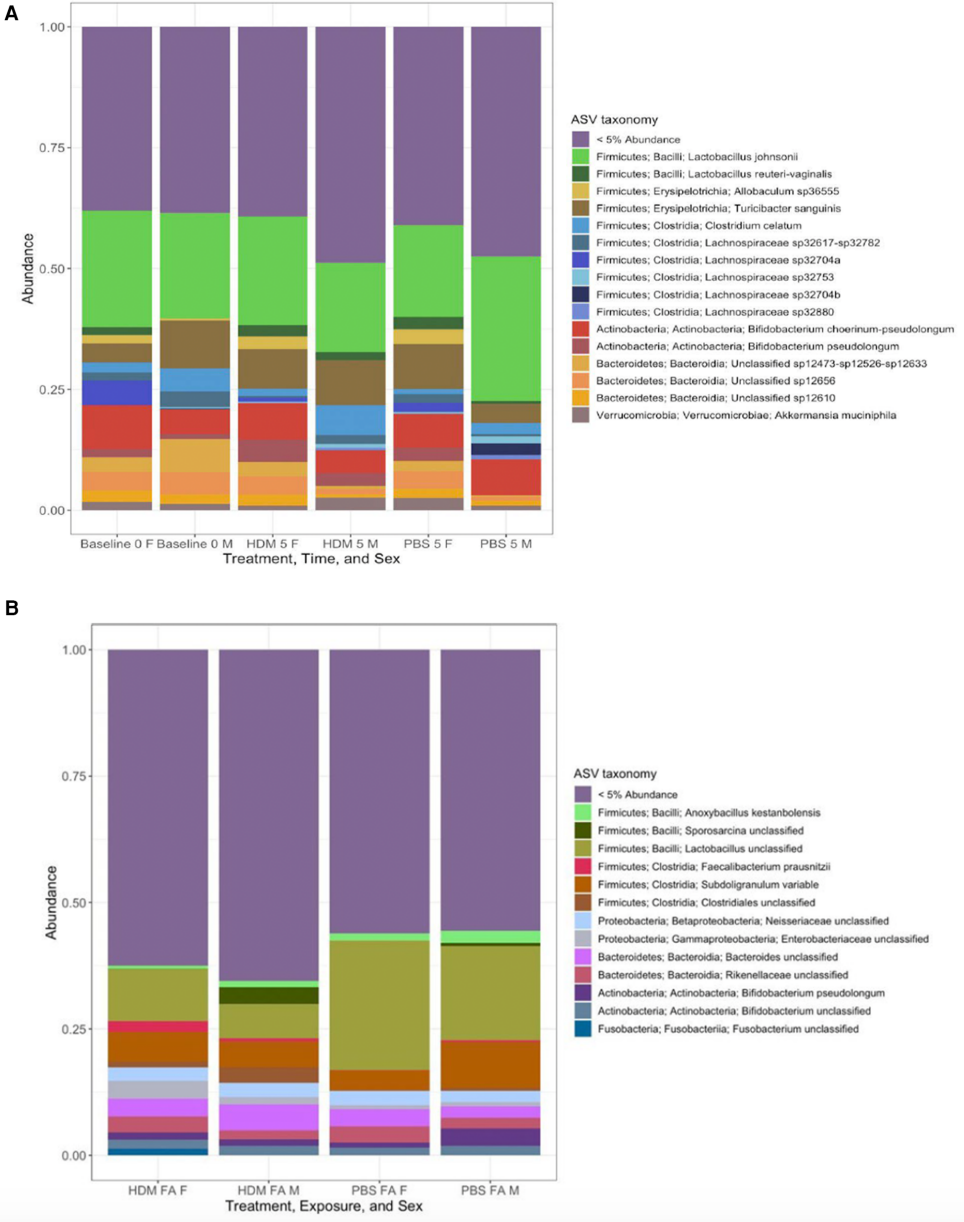
Stacked taxa bar plot of relative abundances of most common gut and lung microbial communities (**A**) ∼18 most common microbial amplicon sequence variants (ASV), selected if they met a threshold of 5% abundance in at least one fecal pellet sample, are displayed in different colors and aggregated together by sample identity (treatment, sex, and time). All other taxa falling below this threshold are grouped together into a ‘rare’ category labeled “<5% Abundance”. (**B**) Samples grouped by “Identity”, deﬁned by their unique combinations of treatment/time, and sex. Each of the bars shows the abundance of named taxa found to be present at least 5% relative abundance in at least one sample of lung tissue. All other taxa falling below this threshold are aggregated into a single “rare” category and labeled “<5% Abundance” (*n* = 2–4/ treatment group).

Among the 16 common microbial amplicon sequence variants (ASVs) that were selected as meeting a threshold of 5% abundance in at least one fecal pellet sample, *Firmicutes* occupied 63%, *Actinobacteria* 13%, *Bacteroidetes* 19%, and *Verrucomicrobia* 6%, of the microbial population in the gut. Among the 13 ASVs that were selected as meeting a threshold of 5% abundance in at least one fecal pellet sample, Phyla- *Firmicutes* occupied 46%, *Proteobacteria, Bacteroidetes, and Actinobacteria* occupied 15% each, while *Fusobacteria* occupied the remaining 8% of the microbial population in the lung. Interestingly, one taxon was found to be shared between the gut and lung microbiome and was present in all sample groups: “Actinobacteria; Actinobacteria; *Bifidobacterium pseudolongum*” as shown in Figure 7.

### 3.7 Bacterial community composition and *Firmicutes/Bacteroidetes* ratio (F: B)

**Figure 8:**
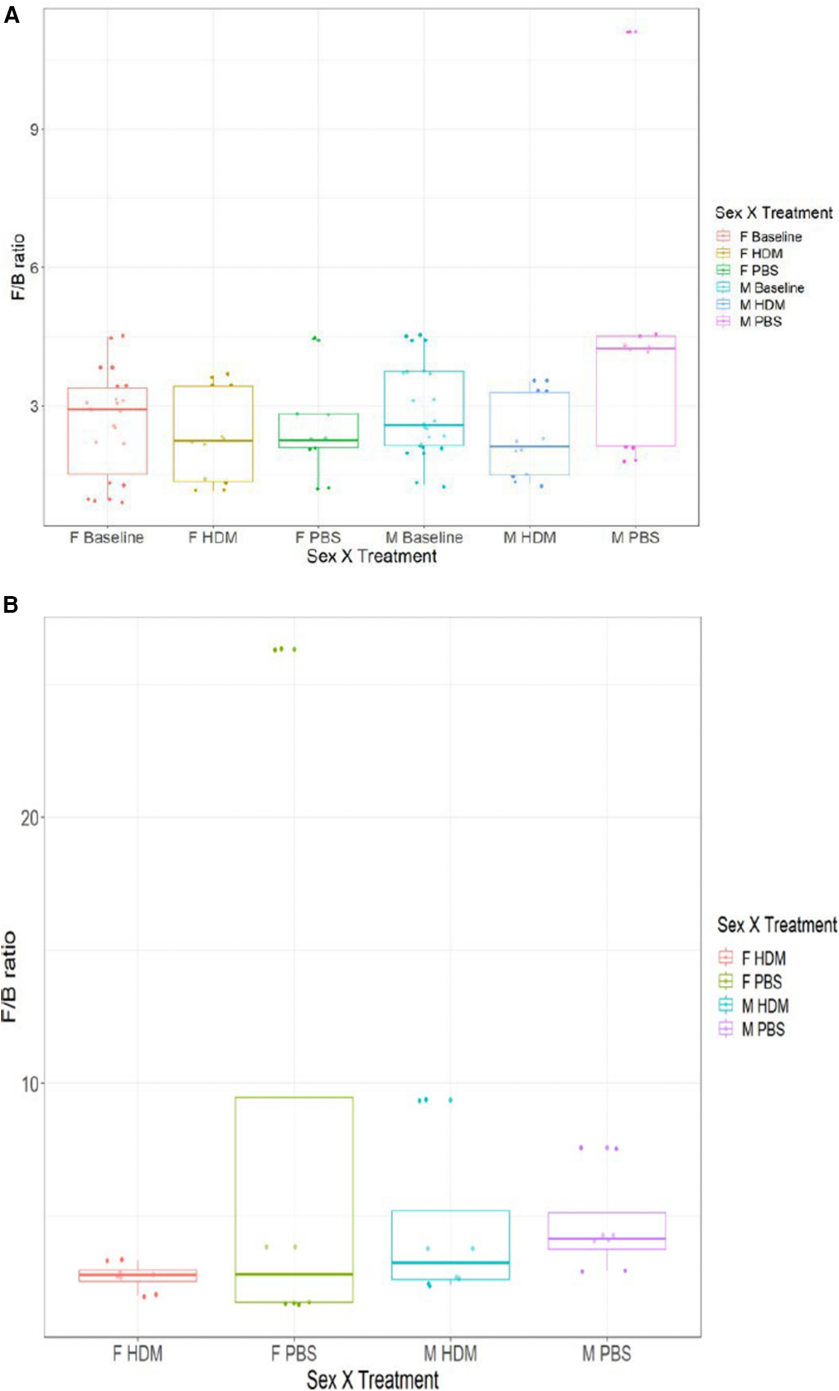
*Firmicutes* to *Bacteroidetes* ratio of 16S gut and lung microbiome among the groups. (**A**) The average ratio of *Firmicutes* to *Bacteroidetes* by absolute abundance found in the gut of male and female samples at week 0 and week 5 (HDM or PBS treatment). (**B**) The average ratio of *Firmicutes* to *Bacteroidetes* by absolute abundance found in the lungs of male and female samples after 5 weeks of treatment (HDM or PBS) (*n* = 2–4/ treatment group).

The pre-exposure and after-exposure F:B in the gut and lungs of the HDM-challenged and control groups are shown in Figure 8. Pre-exposed males had an average gut F:B ratio of 2.88 while the females had an average gut F:B of 2.62. These are the presumed basal levels for each sex in this model. After treatment, the male gut F:B ratio decreased to 2.31 in the HDM-challenge group and increased to 3.00 in the control mice, while female gut F:B ratios decreased to 2.34 and 2.56, respectively following HDM and PBS challenges (Figure 8A) In the lungs, the male PBS group showed an F:B ratio close to 4.00, while all other groups had ratios between 2.00 and 3.00 (Figure 8B).

### 3.8 Alpha and beta diversity

**Figure 9:**
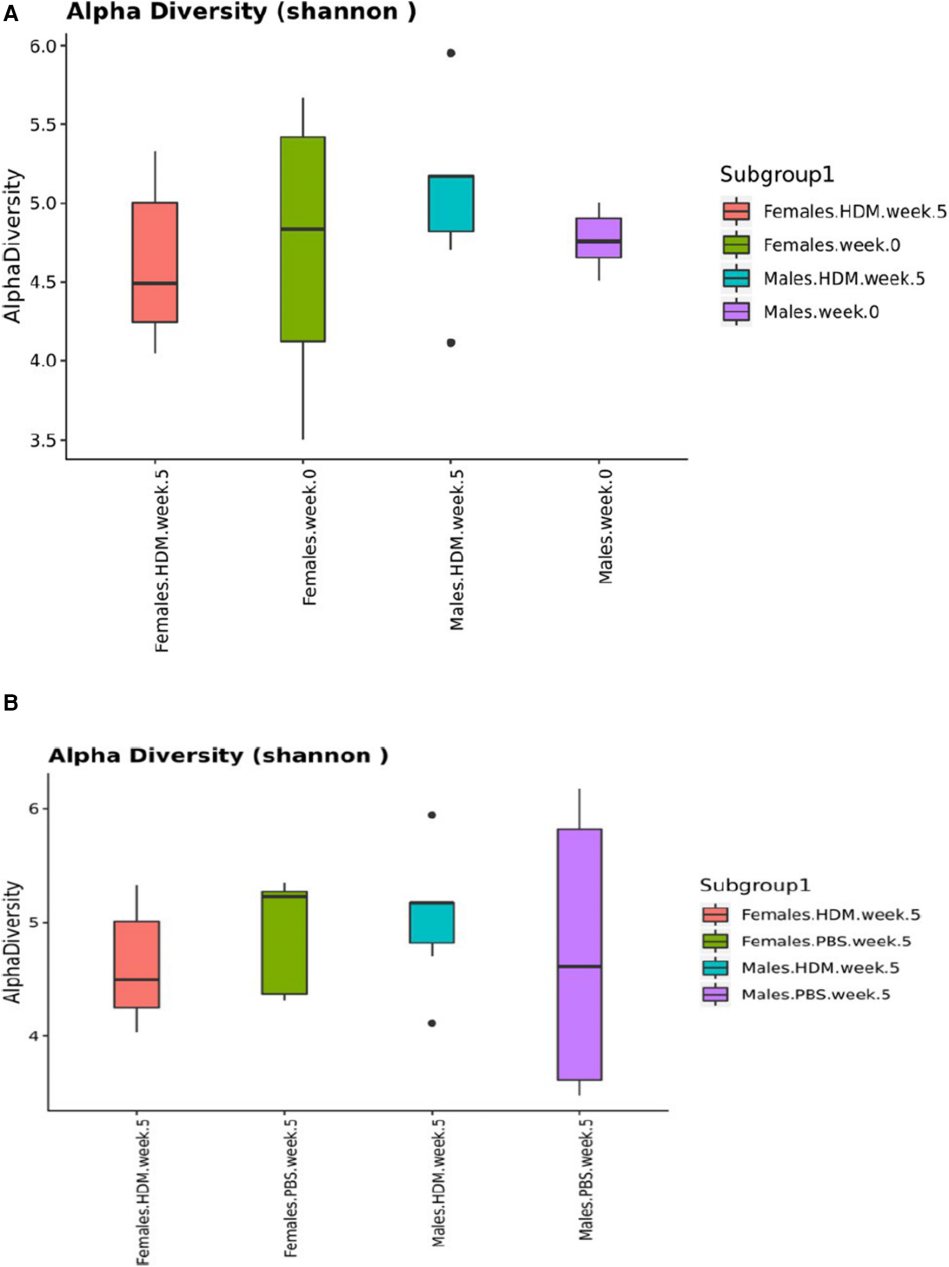
Shannon diversity Index plots of 16S gut and lung microbiome. Box-and-whisker plots depicting the diversity of bacteria in each fecal pellet sample (**A**) and each lung sample (**B**) grouped by identity variables (sex, treatment, and time). This indicates the number of different bacteria in each fecal pellet sample, while accounting for uniformity (*n* = 2–4/ treatment group).

Alpha diversity analysis at week 0 revealed that males and females have similar median values of roughly 4.8 and 4.7, respectively, and that females also displayed a much larger variance (Figure 9). After 5 weeks of HDM challenge, the median value for males increased to about 5.0 with an extreme outlier on both high and low ends, while the median value for females decreased to 4.5. After 5 weeks the median value for PBS-treated males slightly decreased to about 4.7 with a large variance, while the median value for females increased to a value of roughly 5.1.

**Figure 10:**
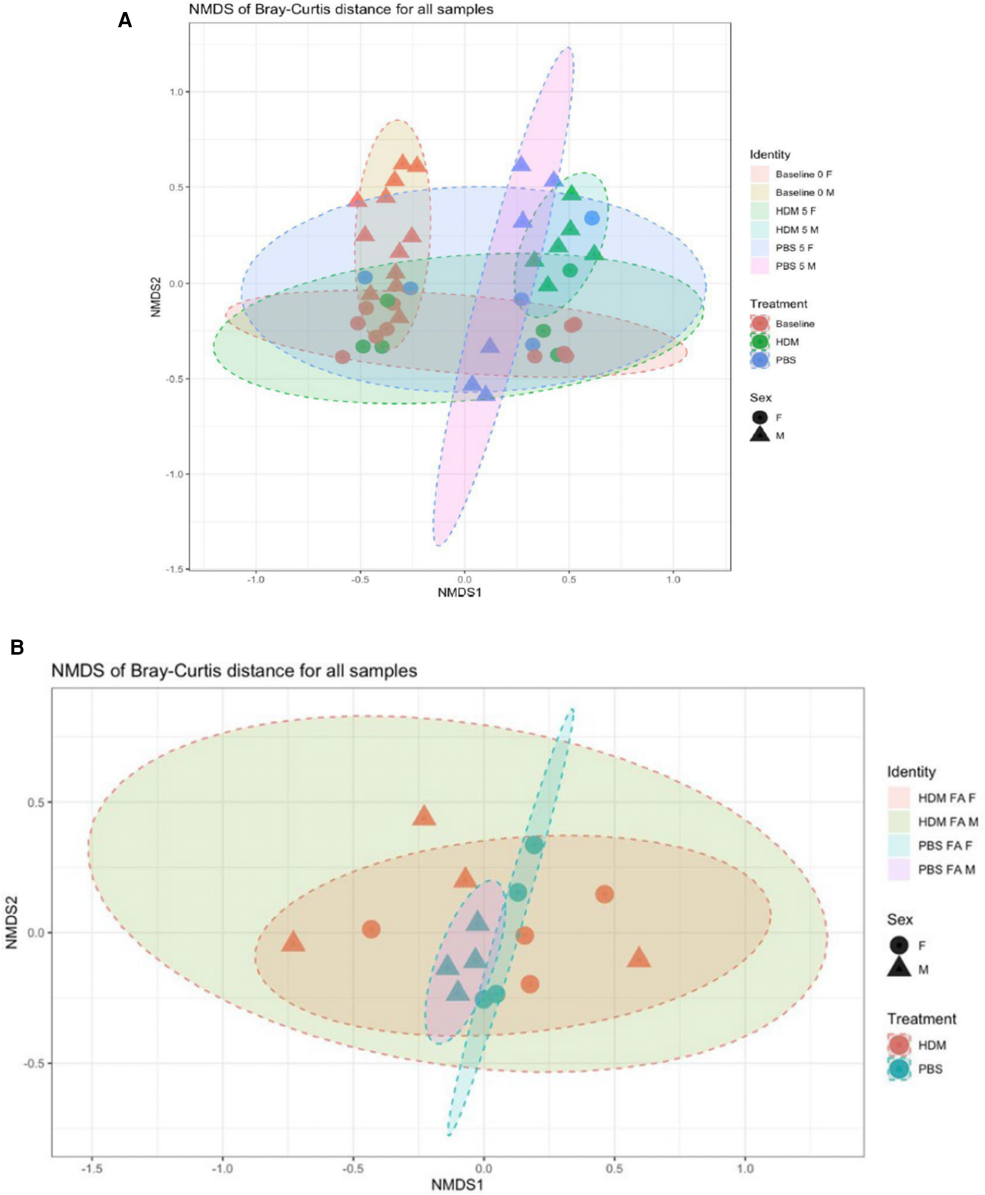
Bray-Curtis nonmetric multidimensional scaling (NMDS) plot of 16S gut and lung microbiome. Each point represents a separate fecal sample (**A**) and lung sample (**B**) Points have symbols representative of their identity (sex, treatment, time) and are overlaid with each other to form visual regions of clustering that represent the similarity/dissimilarity of the samples (conﬁdence Level = 0.90; a = 0.20, *N* = 2–4/ group).

Ordination for the fecal samples by beta diversity (Figure 10A) showed clustering of male baseline, while male week 5 HDM and PBS appear to cluster next to each other. From this analysis, it can be thus assumed that the difference in variation between week 0 and week 5 is leading to a significant time effect in the gut PERMANOVA tests. On the other hand, ordination for the lung samples (Figure 10B) showed that HDM treatment doesn't necessarily shift the community structure but rather causes samples to spread in this model. It can be assumed that the difference in variation between the treatments leads to a significant treatment effect in the lung's PERMANOVA tests. This illustrates microbiome dysbiosis in HDM-treated animals when compared to the tight, similar communities of the control (PBS) animals. As indicated in Supplementary Tables 1A,B, there was a non-significant effect of treatment on Bray-Curtis similarity between fecal pellet samples when baseline values are included and excluded (*p* = 0.071 and 0.303, respectively); meaning tha the different treatments (HDM or PBS) do not have significantly different microbial community structures, irrespective of sex or time. Considering both sex and treatment together (Supplementary Tables 1C,D), treatment was still not predictive of significantly different microbial communities with (*p* = 0.068) or without the baseline samples (*p* = 0.260). However, sex was significant in both cases (*p* = 0.047 and 0.018) accounting for differences in community structure between samples. Furthermore, when sex, treatment, and their interaction are considered (Supplementary Table 1E), sex alone significantly influences community similarity when baseline values are included (*p* = 0.047) and excluded (*p* = 0.015). Though, the sex by treatment interaction was not significant in either case (*p* = 0.071 and *p* = 0.153). Finally, the variable of time (i.e., week 0 to week 5) had a significant influence on community similarity (*p* = 0.033), as expressed in Supplementary Table 1G.

Conversely, PERMANOVA tests of lung tissue samples described a significant (*p* = 0.003) treatment effect on Bray-Curtis similarity between samples (Supplementary Table 2A); the different treatments resulted in significantly different microbial community structures, irrespective of sex or exposure. Sex (Supplementary Table 2B) was not found to be significant (*p* = 0.397), as was the treatment by sex interaction (*p* = 0.316); therefore, they do not explain differences in community structure between samples.

## 4 Discussion

In this study, we examined the extent to which chronic exposure to a mix of *D. pteronyssinus*, and *D. farinae* HDM in a C57BL/6J mouse model caused sex-specific alterations to the gut and lung microbiomes. Male and female adult C57BL/6J mice were exposed intranasally to either an HDM mix allergen challenge or a PBS control challenge daily for 5 weeks (*n* = 4–6). A prior study observed that allergic airway disease phenotype was induced in female C67BL/6 mice after only 2 weeks, or 5 weeks, of exposure to HDM (33). This was evident by elevated eosinophilia, peribronchial and perivascular inflammation, and airway resistance, which reverted when treatment lasted for 11 weeks (33). More recently, Mostafa et al. reported sex- and strain-specific differences in eosinophilia, gene expression, and pro-inflammatory cytokine expression after only 2 weeks of exposure to HDM (34). In our 5-week model, we observed a severed AHR in male mice compared to the female mice. Similar models have demonstrated that chronic exposure to HDM causes varied respiratory system responses which are time and strain-dependent. For example, C57BL/6J shows higher resistance to AHR compared to other strains of mice and is attributed to their genetic makeup (35–38). Several studies have already reported that females are susceptible to airway inflammation following allergen exposure (39–45), while others have seen higher AHR in males than females (46–48). The greater response to methacholine challenge seen in the males was associated with differential responses in the airway smooth muscle (46, 48).

Sex dimorphism in HDM-mediated airway inflammation has also been reported. Most studies have reported greater infiltration of eosinophils in females than males exposed to allergen (36, 44). In this study, HDM-mediated neutrophil accumulation was greater in females than in males, suggesting that females had severed inflammation compared to males. Mostafa et al. reported higher HDM-eosinophils in males than in females and suggested that the immune cell population in the lungs following allergen exposure was strain-dependent (34). However, this claim was countered by the non-sex-specific study of Boucher et al.*,* where a similar increase in the infiltration of inflammatory cells to the airway of two different strains of mice after HDM exposure was observed (49). Berndt et al. also observed a non-sex specific time-of-day dependent elevation in neutrophils in the acute HDM-exposed group compared to the control group (35). Male and female animals treated with lipopolysaccharide showed a higher neutrophil count compared with control, but no sex differences were observed (50, 51). In human studies, a non-sex specific neutrophilia was seen in the sputum and BALF of asthmatics (52, 53) which was hypothesized to be mediated by IL-17A (54). Interestingly, the ENFUMOSA cross-sectional studies have highlighted that men are four times less likely to suffer from chronic severe airway diseases than women (55). A recent study reported a similar finding, showing an increase in lymphocytes observed in the female HDM-treated group than in males (56). While Fuseini et al. reported a greater increase in lymphocytes, macrophages, eosinophils, and neutrophils in female BALB/C mice compared to males (57), we only observed a greater increase in neutrophils and lymphocytes in females. The differing outcomes may be due to the choice of strain, the concentration of the HDM used, and/or the duration of the protocol adopted to challenge or sensitize the animals.

Genes known to increase immune responses, *Tnfrsf17, Trim71, Cerk, Ccl11, Il12b,* and *Grn* (58–61), were upregulated in the HDM-treated female mice over the PBS-treated group, while only one gene, *Npm1* (62), known to decrease immune response was upregulated in the same group. Interestingly, among the upstream regulators shown by IPA in the HDM-treated males over that of the females was Tp53, a gene implicated in the susceptibility to respiratory diseases (63). Tnf and beta-estradiol were inhibited in the male HDM-treated animals over the females. These two genes are positively related to neutrophilic-inflammation (64–66), suggesting reasons for the reduced inflammation observed in HDM-treated males. *Vegf-a* increased significantly in asthmatics (67, 68), and induced airway inflammation and remodeling in murine mice (69). Also, *Hif-1α* expression was increased after 1 and 5 weeks of exposing mice to allergens (70), whereas *Hif-1α* regulates the expression of the gene that encodes *Vegf-a* (71). One important limitation of this study is that all the data reported comes from sequencing of whole lung tissue homogenates rather than sorting out specific immune cells. Future studies should consider assessing cell-specific gene expression following challenge, as well as their associations with lung and gut microbiome changes.

Our analysis revealed that while the phyla *Firmicutes* and *Bacteroidetes* were predominant in the gut microbial community, in the lung microbial community the phyla that were predominant were *Firmicutes, Proteobacteria, Bacteroidetes*, and *Actinobacteria*. This was in line with prior reports from other researchers (17, 72, 73). The percentage of abundance of these microbial populations varied in the different groups. Previous studies found a varying composition in gut microbiome in early life due to diversity, complexity, and dominant bacterial taxa. Ege et al. named environmental exposures one major microbiome composition influencer (74). *Actinobacteria* has been known to colonize the lung in the early stage of life which is thought to protect the individual against allergy (75) and men are believed to possess it more than women which might account for the severe symptoms of asthma in women. Alaskha Alhamwe et al. observed an elevated proinflammatory immune response following repeated administration of *Acinetobacter Iwoffi*, proving the protective role of the bacteria on the immune system, although the sex of the animals used was not specified (76). Consistent with the previous study, in the gut microbial composition, we observed that the abundance of *Actinobacteria* was reduced in the male HDM and PBS group when compared to the male pre-exposure group (baseline 0week) whereas it was higher in the lungs of the male PBS group compared to the other groups. The abundance of *Proteobacteria* has been linked to the exacerbation of asthma (77), and it was higher in the lungs of the female HDM group compared to the other group. Chiu et al. found a reduction in the abundance of *Firmicutes* in children with asthma and related it to increased asthma risk (78). Interestingly, this agreed with our study as we found it lower in both male and female HDM-challenged groups when compared to the other groups. Recently, the probiotic species, *Bifidobacterium* was revealed as an anti-inflammatory promoter in clinical studies (79), and was found reduced in the male mice in both the PBS and HDM-challenged groups in this study. Furthermore, most studies have demonstrated that *Bacteroidetes* exhibit a pro-inflammatory effect, which may also contribute to the severity of asthma in women than in men, as it was found to be lower in male HDM and PBS groups compared to the female groups.

The *Firmicutes/Bacteroidetes* ratio is a measure of the abundance of the two major phyla of the gut, where an increase or decrease in the ratio is considered dysbiosis (80), it is significantly relevant in the microbial composition of human guts (81). In the gut of the male and female mice, this ratio was decreased following the HDM challenge when compared to the pre-exposure and PBS-challenged groups. In the lung, F: B was decreased in the male HDM vs. PBS group but not in the females. Previous studies reported that F: B evolves from early childhood to adulthood (82).

Alpha diversity refers to the diversity within a single microbial community, typically measured by calculating the number of different microbial taxa (species, genera, etc.) present and the relative abundance of each taxon (83). Alpha diversity is important because it can provide insights into the stability and resilience of a microbial community, as well as its functional capacity (84, 85). The Shannon Diversity Index plots in Figure 6, indicate the number of different bacteria in each fecal pellet sample while accounting for uniformity. The higher the value, the greater the community diversity (86). Bisgaard et al. reported that reduced bacterial diversity is inversely proportional to the risk of allergic sensitization at the first 6-years of life (87). Shannon index was used to measure bacterial diversity within the different samples in this study, which has also been used in similar research (88). We observed dysbiosis in this study, especially in the gut microbiome. The dysbiosis occurred with time, treatment, and sex, and this was associated with the development and exacerbation of allergic asthma (80). We found that the alpha diversity median value increased in the male HDM group and decreased in the female HDM-challenged mice. Interestingly, this observation was reversed in the PBS group.

Scientists have reported higher richness of lung microbiota diversity in asthmatics vs. non-asthmatic patients (13). While some have reported lower richness in the asthmatics compared to control subjects (89, 90), others reported no significant difference between these groups (91, 92). Beta diversity i.e., the differences in microbial community composition between different samples or environments has been used by other researchers (93, 94). These measures compare the presence and abundance of microbial taxa between samples and can provide insights into the factors driving microbial community differences, such as diet, host genetics, and environmental conditions (93, 94). In prior studies, beta diversity, not alpha diversity was significantly different between genders (95).

In summary, our study revealed sex differences in the gut microbial composition in the PBS and HDM-challenged groups, as well as sex-specific inflammatory phenotypes in response to allergic challenge. Researchers have observed sex disparities in the gut microbial composition both in clinical (96) and animal studies (97–100) following ozone exposure, and associated these with differential AHR and inflammatory phenotypes. The microbiome was shown to be sex-biased, and the females were highly diverse compared to the males at puberty (101) in an autoimmune disease context. Most sex-based studies have only considered the gut microbiome leaving the lung microbiome. Few studies, like the ovalbumin (OVA)-induced model of allergic airway inflammation research of Roggenbuck et al., reported a sex disparity in the microbial composition of the lung (102). The current study adds to our body of knowledge about the potential interaction of lung and gut microbiomes in mediating inflammatory and physiological responses to an HDM mix-induced allergic challenge in the lung.

## 5 Conclusion

In conclusion, our findings indicate that the gut and lung microbiome experience sex-specific changes in composition and structure due to chronic exposure to an environmental allergen. These may suggest a contribution of the lung-gut axis in mediating inflammatory mechanisms underlying asthma, and thus contribute to our understanding of sex-specific asthma phenotypes.

## 6 Perspectives and significance

Sex disparity has been established in respiratory disease; hence it becomes important to highlight sex-specific changes in respiratory disease pathophysiology drivers such as microbiome to identify novel sex-specific therapeutics for disease management. This present study investigated the sex-based alterations in the gut and lung microbiome following allergen exposure. Our findings from this study revealed a sex-specific change in the gut microbial composition while, the lung microbial composition did not display sex-specific alterations and only experienced treatment-induced alterations. Further studies are needed to investigate other drivers of sex disparities in respiratory diseases such as the sex chromosomes and the sex hormones.

## Data Availability

The datasets presented in this study can be found in online repositories. The names of the repository/repositories and accession number(s) can be found in the article/Supplementary Material.
