# Supplementary material for: Sex-specific alterations in the gut and lung microbiome of allergen-induced mice
Source: Front Allergy. 2024 Aug 15;5:1451846. doi: 10.3389/falgy.2024.1451846 (PMC11358121; doi:10.3389/falgy.2024.1451846)
Supplement: Supplementary file 1 [file Table1.docx]

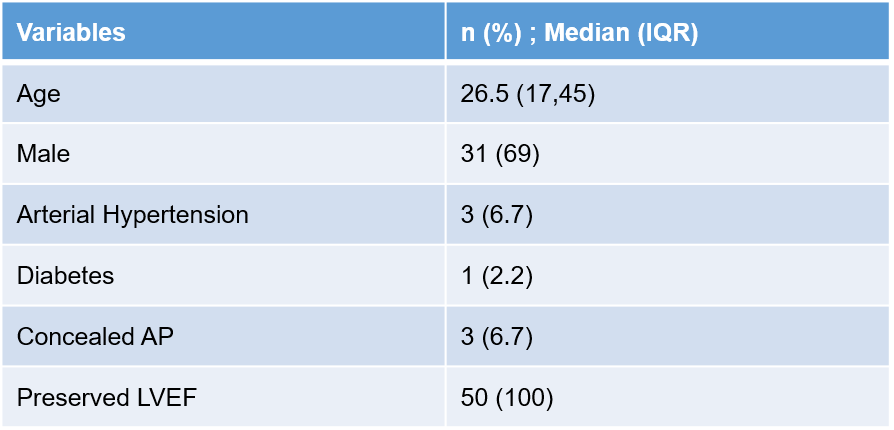


**Table 1**. Baseline population characteristics

**Table 1**. Baseline characteristics are expressed as number and relative percentages or median and Interquartile Range. AP accessory pathway; LVEF left ventricular ejection fraction.
