# Supplementary material for: Sex-specific alterations in the gut and lung microbiome of allergen-induced mice
Source: Front Allergy. 2024 Aug 15;5:1451846. doi: 10.3389/falgy.2024.1451846 (PMC11358121; doi:10.3389/falgy.2024.1451846)
Supplement: Supplementary file 2 [file Table2.docx]

Supplementary Table 2: **PERMANOVA testing for significant differences in ASV abundance in 16S lung microbiome samples between variables (treatment/sex)**

Permutational Multivariate Analysis of Variance tests based on Bray-Curtis similarity of sex, and treatment variables between lung tissue samples. (P ≤ 0.05)

1. Treatment alone

|  | **Df** | **SumOfSqs** | **R2** | **F** | **Pr(>F)** |
| --- | --- | --- | --- | --- | --- |
| Treatment | 1 | 0.948 | 0.207 | 3.647 | 0.003 |
| Residual | 14 | 3.638 | 0.793 | NA | NA |
| Total | 15 | 4.586 | 1.000 | NA | NA |

1. Treatment and sex

|  | **Df** | **SumOfSqs** | **R2** | **F** | **Pr(>F)** |
| --- | --- | --- | --- | --- | --- |
| Treatment | 1 | 0.948 | 0.207 | 3.645 | 0.003 |
| Sex | 1 | 0.258 | 0.056 | 0.992 | 0.397 |
| Residual | 13 | 3.380 | 0.737 | NA | NA |
| Total | 15 | 4.586 | 1.000 | NA | NA |

C. Treatment by sex

|  | **Df** | **SumOfSqs** | **R2** | **F** | **Pr(>F)** |
| --- | --- | --- | --- | --- | --- |
| Treatment | 1 | 0.948 | 0.207 | 3.663 | 0.003 |
| Sex | 1 | 0.258 | 0.056 | 0.997 | 0.393 |
| Treatment:Sex | 1 | 0.275 | 0.060 | 1.064 | 0.316 |
| Residual | 12 | 3.105 | 0.677 | NA | NA |
| Total | 15 | 4.586 | 1.000 | NA | NA |
